# The role of general practitioners in German cancer care: a scoping review

**DOI:** 10.1186/s12875-026-03541-w

**Published:** 2026-09-02

**Authors:** Anne Werner, Julia Katzur, Anne Schrimpf, Jutta Bleidorn, Markus Bleckwenn

**Affiliations:** 1 Institute of General Practice, Faculty of Medicine, Leipzig University, Leipzig, Germany; 2 Institute of General Practice and Family Medicine, Jena University Hospital, Friedrich Schiller University Jena, Jena, Germany

**Keywords:** Cancer, Primary care, Interprofessional collaboration, Integrated care, Patient-centered care, Germany

## Abstract

**Background:**

General practitioners (GPs) play an important role in the care of patients with cancer across the entire cancer continuum, from diagnosis to palliative care. Despite their broad range of responsibilities, structures to facilitate the systematic involvement of GPs into cancer care remain insufficient in Germany. To date, the specific role and contribution of GPs within the German cancer care system have not been comprehensively synthesized.

**Objective:**

This scoping review aimed to summarize the available evidence on GP involvement in cancer care in Germany, including their roles across the cancer trajectory, collaboration with other healthcare providers, and barriers and needs affecting their participation in cancer care.

**Study selection and analysis:**

A scoping review was conducted according to Arksey & O’Malley. The search was conducted on 2 January 2024 in the databases Cochrane Library, MEDLINE, and Web of Science. Eligible studies examined the role of GPs in the care of patients with cancer or long-term cancer survivors in Germany. Extracted review’s domains (GPs’ roles and responsibilities, collaboration, needs) were derived from an exploratory literature search and a GP advisory board. Findings were synthesized narratively. Studies published before 2008 were excluded.

**Results:**

The search yielded 646 hits, of which 71 could be included in the full-text screening and 24 in the data extraction. GPs are involved in all phases of cancer care, including diagnosis, active treatment, follow-up care, and palliative care. GPs as “persons of trust” and experts in counselling, provide patient-centered cancer care and emotional support. GPs collaborate with other healthcare providers, but are rarely involved in specific treatment decisions. Deficiencies in communication, information exchange, and role clarification were identified. GPs expressed a need for greater involvement and better information through informal, bidirectional communication with various stakeholders in the oncology healthcare sector. Evidence on GP involvement in survivorship care and the management of comorbidities in patients with cancer was limited.

**Conclusions:**

Efficient structures for information exchange and knowledge transfer between GPs and other healthcare providers may support continuous and comprehensive care for patients with cancer. Future guidelines should explicitly address the role of GPs and provide clear recommendations for their integration into cancer care pathways.

**Scoping review registration:**

https://doi.org/10.17605/OSF.IO/T8XEN.

**Supplementary Information:**

The online version contains supplementary material available at https://doi.org/10.1186/s12875-026-03541-w.

## Introduction

In 2020, approximately 1.6 million people in Germany were living with a cancer diagnosis made within the last five years [1, 2]. The number of cancer survivors continues to rise steadily, driven by population ageing and advances in medical care that have improved survival rates [3]. Consequently, patients with cancer and survivors have to cope with both the effects of the disease and the long-term consequences of its treatment [3].

In Germany, most residents regularly consult a general practitioner (GP) [4]. According to the World Organization of Family Doctors (WONCA), general practice is characterized by a holistic, person-centered approach [5]. In particular, GPs address both physical and psychological health problems while taking into account patients’ social, family, and cultural environments [5]. This approach is particularly relevant in cancer care, where patients often face complex medical and psychosocial challenges [5]. In contrast to specialists, who typically focus on the pathology itself, GPs integrate medical conditions with patients’ beliefs, behaviors, concerns, and wishes [5].

Cancer care is inherently interdisciplinary and requires close collaboration among multiple healthcare professionals. However, deficiencies in collaboration and loss of information during transitions between healthcare providers may negatively affect patient outcomes [6]. This presents a major challenge for all healthcare providers involved, but particularly for GPs. As part of their role, GPs support patients by coordinating care, managing interfaces with other specialties, and, when necessary, acting as advocates for their patients [5, 6]. It is further widely recognized that GPs contribute to multiple stages of the cancer continuum, including diagnosis, treatment of symptoms, management of long-term and treatment late-effects, as well as in palliative care [7, 8]. However, most health services research primarily focused on treatment-related outcomes, such as costs, access, and quality, with limited attention given to the integration of GPs in cancer care [9].

In Germany, effective collaboration in cancer care is often hindered by unclear roles and responsibilities [10]. The primary objective of this Scoping Review was therefore to provide a comprehensive overview of the multifaceted involvement of GPs in the care of patients with cancer and long-term cancer survivors in Germany. Specifically, we explored the roles of GPs across different stages of the cancer trajectory, and their overarching contribution to cancer care, which is grounded in the professional identity and core principles of general practice. In addition, we examined the existing structures and potential challenges in cross-sectoral collaboration between GPs and other healthcare providers involved in cancer care in Germany. Particular attention was given to identifying barriers and needs faced by GPs in Germany in providing primary care to patients with cancer. The term *integration* was used as an umbrella concept describing the extent to which GPs are incorporated into cancer care across different levels of the healthcare system. This includes their clinical roles and responsibilities throughout the cancer trajectory, their collaboration and coordination with other healthcare professionals, and their organizational and system-level embedding within cancer care structures. Related concepts such as involvement, coordination, collaboration, and shared-care are therefore considered complementary dimensions of GP integration rather than distinct phenomena. Given that the concept of integration is broad and multidimensional, and that the available evidence is methodologically heterogeneous, a scoping review was considered the most appropriate approach. Scoping reviews are particularly suited to mapping diverse bodies of evidence and identifying conceptual, methodological, and empirical gaps in research fields that remain insufficiently defined. This review was guided by the following research questions:

1. What roles do GPs in Germany play in the care of patients with cancer across different stages of the cancer trajectory?
2. How is collaboration between GPs and other healthcare providers in German cancer care described?
3. Which barriers, facilitators, and unmet needs have been reported by GPs and oncologists in the literature regarding the involvement of GPs in cancer care in Germany?

Overall, this scoping review aimed to summarize the existing literature on the complex integration of GPs in cancer care in Germany, identify gaps in both the evidence base and current care provision, and highlight future research priorities. A deliberately broad scope was chosen to capture the wide range of factors influencing GP involvement in oncology care and to reflect the heterogeneity of the available literature. Consequently, the review incorporates perspectives not only from GPs themselves but also from other healthcare professionals and the wider healthcare system. In addition, the review aimed to serve as a reference for future research to improve the integration of GPs in the care of patients with cancer in Germany.

## Methods

This scoping review followed the methodological framework of Arksey & O’Malley [11]. The PRISMA Extension for Scoping Reviews (PRISMA-ScR) was used for reporting [12]. The review protocol was registered on the Open Science Framework on December 19, 2023 [13].

### Eligibility criteria

No restrictions were placed on study design, publication type, cancer entity, or stage of disease. Given the exploratory nature of a scoping review, a broad range of evidence sources was considered to comprehensively map the available literature. No language restrictions were applied.

Our review included studies that described the roles and responsibilities of GPs in the care of patients with cancer or long-term survivors within the German healthcare system. We also considered studies reporting on interdisciplinary collaboration, physicians’ needs, and patient characteristics. We included studies in which GP-related aspects constituted the primary focus, as well as studies in which GP involvement was not the main topic but where relevant information could be clearly identified and extracted (e.g., through distinct specific items, interview quotations, or stratified analyses).

Studies reporting exclusively on patients’ perspectives of the GP’s role in cancer care were excluded. For studies reporting mixed perspectives (from patients and physician), we extracted only findings related to healthcare providers. This decision was made to maintain conceptual clarity and to avoid conflating provider-focused and patient-focused evidence. The latter will be addressed in a separate review. We also excluded studies that were older than 15 years, as substantial changes in cancer care, the healthcare system, and the role of GPs were expected during this period.

### Search strategy and information sources

We used English search terms based on conceptualizations of the term GP or family medicine (A) and cancer (B). Search terms were developed using established terminology for these concepts and were informed by discussions with patients and stakeholders regarding GP involvement in cancer care.

To ensure transparency and reproducibility, the complete search strategies for all databases (MEDLINE, Cochrane Library, Web of Science) are provided in Supplementary File 1. Searches were conducted using database-specific search fields, including Title/Abstract searches in MEDLINE, Topic searches in Web of Science, and full-text searches in the Cochrane Library. Apart from the predefined 15-year publication window, no additional filters or language restrictions were applied.

During pilot searches, both English and German search terms were tested. However, all studies identified through the German-language searches were also retrieved using the English-language search strategy, wherefore German search terms were not included in the final search strategy. Consequently, German-language articles identified through the English-language searches were eligible for inclusion.

Consistent with established scoping review methodology, we employed a deliberately broad, concept-based search strategy to capture the diverse and multifaceted aspects of GP involvement in cancer care.

We searched the following databases: MEDLINE (Pub Med), Cochrane Library, and Web of Science. The searches were conducted on 2 January 2024 (Table 1).

**Table 1:** Search strategy (e.g. Pubmed)

TiAb: A AND B AND C	
A	“general practitioner*” OR “family physician*” OR “family doctor*” OR “primary care” OR “family medicine”
B	cancer OR oncolog* OR tumor
C	german*

### Article selection

**Fig. 1:**
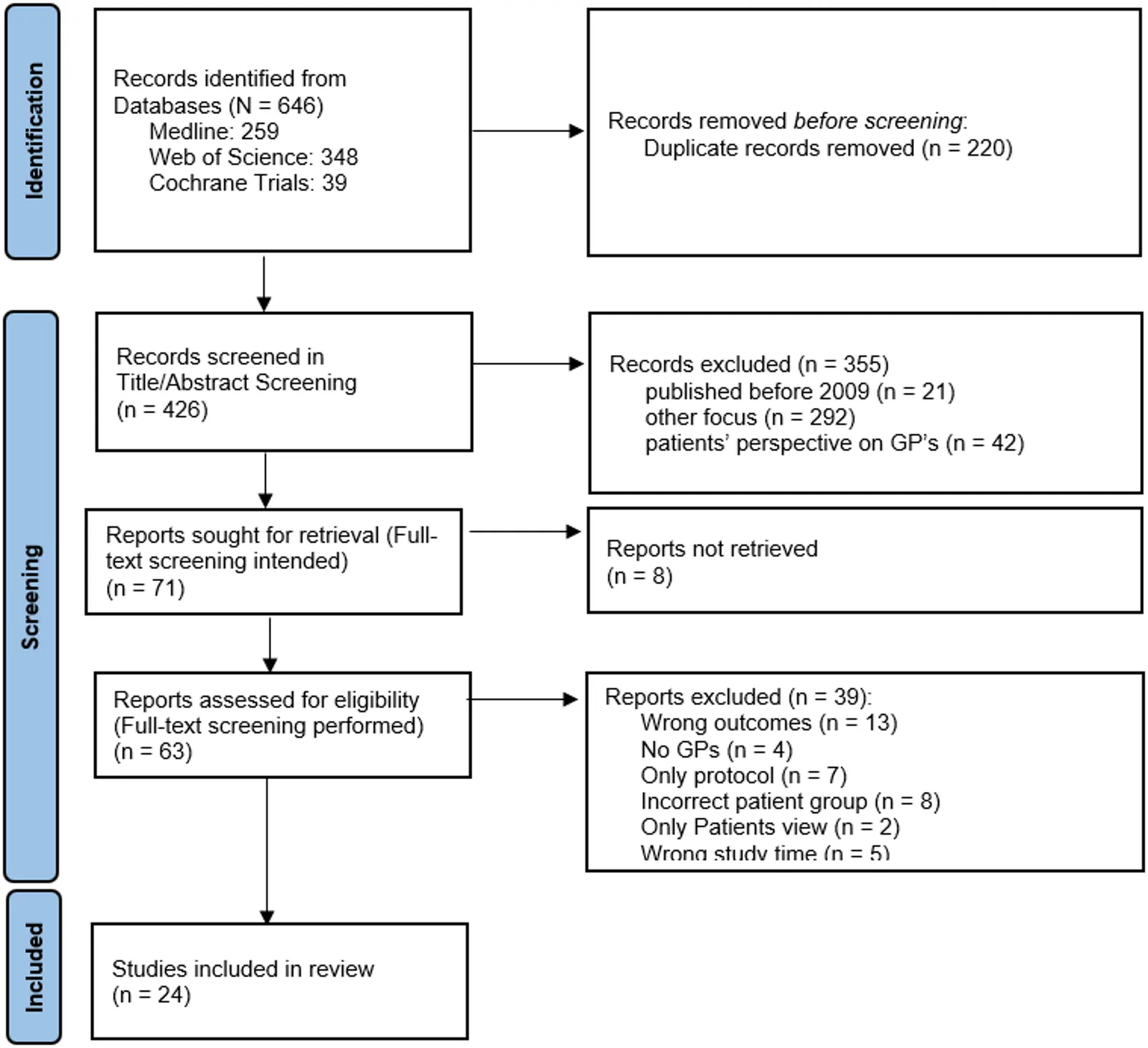
Flow chart of the study selection process

All articles identified through the systematic literature search were transferred to Rayyan for screening [14]. The screening process was conducted in successive stages: First, duplicates were removed by AW and a research assistant (JO). Subsequently, AW and JK independently screened the titles and abstracts of the retrieved articles. Articles considered potentially relevant were transferred to Microsoft Excel for full-text assessment. Full-text screening was performed independently by AW and JK. Discrepancies regarding study eligibility were resolved through discussion between AW and JK. When consensus could not be reached, AS was consulted to provide a final decision. A flow chart illustrating the identification of relevant articles is presented in Fig. 1.

### Data extraction and narrative synthesis

The data extraction framework was informed by preliminary literature searches conducted prior to the review, a focus group as part of the GP advisory board of the Institute of General Practice at Leipzig University, and the clinical experience of JK as a practicing GP. Data extraction was conducted independently by two reviewers (AW and JK) who each extracted data from half of the included articles and cross-checked the data extracted by the other reviewer. To ensure consistency, data extraction procedures and coding rules were developed by consensus and documented in a protocol accessible to all members of the research team.

For each included study, the following information was extracted:

1. Study characteristics (region, sample size, methodology, data analysis);
2. Characteristics of patients/survivors (cancer entity, disease stage, comorbidities);
3. Characteristics of physicians;
4. GP roles and responsibilities (diagnosis, management of patients with acute cancer, treatment of comorbidities, palliative care, social-medical issues, support for relatives, physician-patient communication);
5. Interdisciplinary collaboration (general aspects, satisfaction among providers, formalization);
6. Information sources used by GPs;
7. Barriers, facilitators, and unmet needs related to GP involvement in cancer care.

Data were analyzed by using a deductive and an inductive approach and synthesized by using a narrative approach. The extracted information was initially compiled in a Microsoft Word document and organized by AW. Each extracted statement (verbatim quotation) and information was referenced and coded according to the predefined extraction categories (deductive approach). Individual quotations or information could be assigned to more than one code. Subsequently, statements and information with similar content were grouped, compared, and synthesized. During this process, recurring subthemes were identified inductively within the predefined deductive categories, leading to the generation of second-order categories that further elaborated the analytical framework. The thematic domains and subdomains presented in the Results section were derived from the categories “GP roles and responsibilities,” “interdisciplinary collaboration,” “information sources,” and “barriers, facilitators, and unmet needs.” These categories were developed to summarize recurring patterns across studies and should not be interpreted as the result of a formal qualitative synthesis. Further details on the guiding questions and procedures used for data reformatting are provided in Supplementary File 2. The categories, subcategories, and overall thematic structure were developed collaboratively and discussed by AW and JK. In accordance with scoping review methodology, no formal critical appraisal or risk-of-bias assessment was conducted, as the primary objective was to map the breadth and nature of the available evidence rather than to evaluate methodological quality. Given the substantial methodological heterogeneity of the included studies, emphasis was placed on identifying overarching patterns and recurring themes across the literature. The implications of this heterogeneity are addressed in the Discussion.

## Results

### Characteristics of included studies

A total of 24 articles were included in this scoping review (Table 2). Detailed information on the content of individual studies can be found in Supplementary file 3. The earliest study was published in 2013, based on survey data collected between 2011 and 2012 [15]. Most studies (*n* = 16) used quantitative methods [16–31]. Fewer studies (*n* = 7) used qualitative methods [15, 32–37] and only one study adopted a mixed-methods design [38]. The included studies varied considerably in terms of data sources and sample sizes. Analyses of health insurance data ranged from 1,747 cases [16] up to 13,111 cases [17]. Results from interview studies were based on information from 3 GPs [36] up to 84 physicians (42 GPs, 42 other physicians) [34]. Questionnaire studies were based on information of 112 hospices [24] up to 1,428 GPs [26]. Database studies analyzed datasets comprising between 1,362 patients [19] and 284,333 patients [21].

**Table 2:** Study characteristics

Category	subcategory	*n*	reference
		24	
Year of publication	2013	1	[15]
	2014	2	[32, 37]
	2015	1	[33]
	2016	2	[18, 19]
	2017	4	[27, 28, 34, 35]
	2018	4	[25, 26, 31, 36]
	2019	1	[16]
	2020	1	[23]
	2021	3	[17, 20, 30]
	2022	2	[24, 29]
	2023	3	[21, 22, 38]
Cancer entity	No specification	16	[15, 18–20, 23–27, 29, 32–35, 37, 38]
	Gastrointestinal	4	[16, 28, 30, 36]
	Breast	4	[16, 21, 22, 28]
	Lung	2	[17, 31]
	Prostate	1	[16]
Region	No specification	19	[17–19, 21–31, 34–38]
	Hesse	1	[16]
	Baden-Wuerttemberg	1	[20]
	5–7 from 16 states	3	[15, 32, 33]
Evidence-source	Insurance data	4	[16, 17, 20, 31]
	Databases of privately operated or commercial data providers	5	[19, 21, 22, 27, 29]
	Questionnaire	6	[18, 23–26, 28]
	Interviews	8	[15, 30, 32–36, 38]
	National cancer guidelines	1	[37]
Disease stage	Acute treatment situation	2	[21, 34]
	Long-term survivor	2	[21, 30]
	Palliative care	4	[15, 16, 20, 24]
	No specification	16	[17–19, 23, 25–29, 31–33, 35–38]
Review domains	GP roles and responsibilities	16	[15, 17–19, 21–25, 27, 29–33, 35, 38]
	Interdisciplinary collaboration	9	[15, 16, 20, 23, 26, 28, 32, 34, 38]
	Barriers, facilitators, and unmet needs for GP involvement in cancer care	8	[15, 18, 25, 32, 34, 36–38]

This review synthesized data from various sources, including GPs [15, 18, 21, 23, 25, 26, 28, 30, 32–34, 36, 38], patient health records [16, 17, 19, 20, 22, 27, 29, 31], oncologists [18, 21, 34, 35], urologists [34], gynecologists [21], medical guidelines [37], hospices [24], and others such as oncology nurses, medical assistants, and social workers [21, 25, 30]. Characteristics of the physicians included in the reviewed studies are presented in Supplementary file 4.

Based on the included studies, evidence was extracted and synthesized regarding the roles and responsibilities of GPs, interdisciplinary collaboration in cancer care, and barriers, facilitators, and unmet needs related to GP involvement. Of the 24 studies included, 15 focused on the role of GPs as the primary focus of investigation [15, 18–20, 23, 25–28, 32–35, 37, 38]. Nine studies discussed the involvement of GPs alongside the involvement of other professionals [16, 17, 21, 22, 24, 29–31, 36].

### GPs’ roles and responsibilities in German cancer care

Information on the roles and responsibilities of GPs in Germany in cancer care was reported in 21 of the 24 included studies.

#### GPs’ involvement across the cancer trajectory

The included studies showed that GPs are involved throughout all phases of cancer care, including diagnosis, active treatment, follow-up and palliative care [15, 17–19, 21–25, 27, 29–33, 35, 38]. In a qualitative study, participating GPs emphasized the importance of maintaining an active role across all stages of cancer care [32]. Three studies, including qualitative interviews with GPs, described that the role of the GP becomes increasingly important during the palliative phase [15, 18, 32].At the diagnostic stage, GPs frequently serve as the first point of contact. One database study found that patients aged 41–70 years were more likely to receive a cancer diagnosis from a GP than patients in other age groups [27]. According to this study, the cancers most commonly diagnosed by GPs included malignancies of the digestive organs, breast cancer, and skin cancer [27]. Similarly, an insurance-based study of patients with lung cancer found that GPs most often made the diagnosis in the outpatient setting [17]. During active treatment, patients with cancer continue to consult their GPs regularly. According to a mixed-methods study, patients attended GP consultations monthly or more frequently during treatment, whereas consultation frequency declined to approximately quarterly visits during the first five years of follow-up care. Most appointments were scheduled according to oncologists’ recommendations [38]. Only three studies addressed GP involvement in specific oncological treatments [21, 23, 29]. Among patients with breast cancer receiving endocrine therapy, GPs initiated treatment with aromatase inhibitors in 7.1% of cases and tamoxifen in 6.6% of cases [21]. Initiation of endocrine therapy by GPs or oncologists, compared with initiation by gynaecologists, was associated with a higher risk of treatment discontinuation [21]. Treatment with tyrosine kinase inhibitors (TKI) was rarely supervised by GPs, as evidenced by only one questionnaire study [23]. Most GPs reported low confidence in monitoring TKI treatment and therefore relied on specialists for treatment-specific decisions and surveillance [23]. Further, one database study reported that GPs are the primary providers of outpatient care for patients with cancer-associated thrombosis, issuing most anticoagulant prescriptions [29]. They treat their patients in accordance with guidelines and in consideration of prognosis [29].

Evidence regarding GP involvement in follow-up and survivorship care was comparatively sparse. Although one study reported that GPs cared for more patients during follow-up than during active treatment [38], no included study specifically focused on GP-led cancer follow-up care.

The strongest evidence for GP involvement was observed in palliative care. An interview study found that patients with advanced cancer consulted GPs more often than oncologists [18]. Another qualitative study described how patients often returned to their GP after specialist treatment options had been exhausted [32]. In a large insurance-based study of patients with lung cancer, particularly those living in rural areas, GP consultations remained common during the final 30 days of life [31]. Similarly, one study reported that GPs were among the physicians most frequently involved (after palliative care physicians) in the care of hospice residents [24]. However, in one qualitative study, GPs reported limitations in managing invasive procedures (e.g., symptom control in dyspnea or severe pain) [15] or other complex medical situations that they cannot manage at home. Participants also reported challenges associated with providing continuous, around-the-clock palliative care, particularly when caring for several patients simultaneously over extended periods, which could compromise other practice responsibilities [15].

Regardless of the cancer stage, symptom management emerged as a central component of GP care [19, 25, 38]. One insurance-based study found that GPs prescribed the full range of analgesics, co-analgesics, and adjuvant medications for cancer-related pain management [19]. Two studies reported that GPs are more likely than oncologists to be consulted about complementary and alternative medicine (CAM). CAM was commonly used to treat fatigue, tumor-related pain, psychological problems such as anxiety and depression, nausea, vomiting, loss of appetite, taste changes, and inflammation of the mucous membranes [18, 25]. An interview study reported that although GPs generally regarded counselling on CAM as part of their professional role, they typically discussed CAM only when initiated by patients and did not routinely inquire about its use [33].

Two studies described that GPs provide counselling on lifestyle-related factors such as diet and physical activity as these can contribute to the recovery process [18, 38].

Despite the high prevalence of multimorbidity among patients with cancer, evidence regarding the management of comorbid conditions was limited. Only five of the 24 studies included reported information on patients comorbidities [17, 27, 29–31]. None of these studies addressed the impact of patients’ comorbidities on cancer care and the specific management of comorbidities by GPs during active cancer treatment. Only one study, involving colorectal cancer survivors, examined the number of GP and specialist visits in relation to patients’ comorbidities [30].

#### GP-patient relationship

##### GPs as “persons of trust”

The role of GPs as trusted physicians emerged as a recurring theme across four qualitative studies [15, 32, 33, 35] and one quantitative study [18]. One qualitative study described the GP-patient relationship as being based on long-standing trust [33]. Two qualitative studies described GPs as “persons of trust” who address anxiety and psychosocial challenges of experienced by patients with cancer and provide them with psychological support [32, 35]. One interview study reported that patients consulted GPs more often than oncologists regarding mental health, survivorship, and psychotherapy support [18].

Some oncologists expressed concerns that GPs in their role as “persons of trust” might provide outdated information or question treatment decisions, thereby affecting the physician–patient relationship established by oncologists [35]. In one qualitative study, GPs emphasized the importance of maintaining hope and trust throughout the disease course. As an example, some GPs counsel their patients about CAM, even when they themselves are not fully convinced of its effects [33]. One qualitative study reported that GPs described a high degree of emotional involvement with their patients [15]. In palliative care, they expressed difficulties coping with the separation from patients as they transition to other settings, such as hospices [15].

##### Person-centered cancer care

Evidence supporting person-centered cancer care originated exclusively from qualitative studies [15, 32, 35]. Two studies described that GPs’ long-standing relationships with patients enabled them to draw on knowledge of patients’ medical histories, comorbidities, and social circumstances when providing person-centered cancer care [32, 35]. GPs reported considering prognosis, multimorbidity, treatment burden, and potential side effects when advising patients about therapeutic options [32]. GPs see their role in preserving quality of life [32] and in providing emotional support, particularly in palliative situations [15].

Oncologists participating in three qualitative studies see the role of GPs as providing an “amicable sphere of caring and life coaching”, while they see themselves in an evidence-based treatment role [15, 32, 35].

##### Communication and counselling

Evidence supporting this theme originated primarily from qualitative studies [15, 32, 33, 35], whereas quantitative studies did not specifically address this aspect. One GP in an interview study described general practice as “spoken medicine” [33]. In another qualitative study, participants emphasized the importance of being a resource for their patients by answering questions that have remained unanswered and helping patients understand their cancer diagnosis, its implications and support them in making informed treatment decisions [32]. Additionally, one qualitative study reported that GPs provide emotional support to families of patients receiving palliative cancer care by talking about the disease, illness-related anxiety, and death, and assisted them in making decisions regarding palliative care services and hospice referrals [15]. In line with this, another qualitative study reported that GPs recognize their patients’ fundamental needs for compassion, support, and empathy, while emphasizing the value of open communication [33]. Participating GPs reported communicating honestly when treatment options are no longer available [33]. Similarly, one qualitative study involving oncologists described GPs as mediators, helping patients comprehend the information discussed during oncologist consultations [35]. This role positions the GP as a bridge between the patient and the specialist to reinforce the treatment proposed by the oncologist [35].

### Interdisciplinary collaboration

#### Collaboration between GPs and specialists

Evidence from both quantitative and qualitative studies indicates that GPs play a central role in coordinating care for patients with cancer and routinely collaborate with specialists across different healthcare settings [15, 16, 20, 23, 26, 28, 32, 34, 38]. Collaboration patterns varied according to the stage of care and clinical context. A study of patients receiving oral TKIs found that GPs in urban areas were more likely to refer patients to organ-specific specialists for therapy monitoring than those in rural areas [23]. In palliative care, GPs collaborated with home-care providers, emergency services, and specialist outpatient palliative care (SOPC) teams [15, 20]. An analysis of insurance data from 9,273 deceased patients with breast, prostate, or colorectal cancer revealed that GPs prescribed SOPC in more than 90% of cases [16].

One study identified substantial differences in collaboration strategies between GPs and organ-specific specialists such as urologists and oncologists [34]. This study reported that organ-specific specialists use reciprocal communication, including formal and informal communication pathways. Formal structures, such as tumor boards, fostered professional relationships and supported informal collaborative structures, such as telephone calls [34]. In terms of content, communication among specialists primarily focused on sharing information on cancer care (e.g., specific treatment decisions and responsibilities) [34]. In contrast, GPs were rarely involved in formal communication pathways, such as tumor boards or multidisciplinary quality circles in palliative care, or in decision-making regarding therapeutic interventions [34]. Instead, GPs relied on unidirectional communication, receiving instructions from different specialists (e.g., concomitant testing, writing prescriptions, communicating diagnoses). The same study reported that GPs frequently acted as coordinators of care by conducting follow-up examinations and monitoring within primary care settings, thereby reducing the need for patients to attend additional specialist appointments [34]. The authors identified a need for collaboration in symptom management, managing comorbidities, communication of diagnoses, organization of palliative home care, and coordinating appointments [34].

#### GPs’ satisfaction with collaboration

GPs’ perceptions of collaboration were addressed in two quantitative [26, 28], two qualitative studies [15, 32] and one mixed methods study [38]. Overall, GPs reported moderate to high levels of satisfaction with interdisciplinary collaboration, although satisfaction varied according to the healthcare provider involved [26]. In this survey, GPs expressed the greatest satisfaction with collaboration involving hospices, SOPC, and inpatient palliative care services [26]. Qualitative findings suggested that collaboration with palliative care specialists was particularly valued because it provided support in complex situations, such as pain management, medication adjustments, and family counselling, thereby reducing the burden on GPs [15]. In contrast, lower levels of satisfaction were reported regarding collaboration with physicians working in large hospitals and with outpatient psycho(onco)logists/psychotherapists. The opinions of GPs were divided on the former, while they were dissatisfied or did not use the latter’s services [26].

Across studies, sufficient and timely communication was linked to GPs’ satisfaction with collaboration [26, 32, 38]. Two studies reported that GPs were dissatisfied when they did not receive discharge letters or telephone calls regarding their patients’ treatment in a timely manner [38]. In some cases, GPs need to actively obtain relevant information by contacting patients or colleagues [32]. One qualitative study found that inefficient care coordination can place a burden on GPs, resulting in inconsistent care and requiring them to spend additional time clarifying treatment plans and explaining information to patients [32]. Conversely, timely access to appointments for patients and confidence in the competence of collaborating healthcare providers were associated with greater satisfaction [26].

Communication quality was perceived to decline with increasing organizational size of the collaborating institution, particularly in larger hospitals [26]. However, a questionnaire-based study of GPs found that they perceived no difference in communication quality between certified cancer centers (breast cancer centers and colon cancer centers) and standard clinics [28]. In addition, GP characteristics appeared to have only a limited influence on overall satisfaction with collaboration, as practice location, years of professional experience, and additional qualifications were generally not associated with substantial differences in satisfaction [26]. However, GPs with additional qualification in palliative care reported lower satisfaction with collaboration involving outpatient oncologists and physicians in small hospitals [26]. Further, GPs working in group practices reported greater satisfaction with collaboration involving hospice/palliative care units [26].

### Barriers, facilitators, and unmet needs for GP involvement in cancer care

Evidence from eight quantitative and qualitative studies identified a range of structural, organizational, and system-level barriers that affect GP involvement in cancer care and emphasized several unmet needs related to interdisciplinary collaboration [15, 18, 25, 32, 34, 36–38].

#### Need for improved interdisciplinary collaboration

A recurring theme was the requirement for improved collaboration between GPs and other healthcare providers involved in cancer care [32, 34, 36]. In two qualitative studies, GPs reported a need for improved information exchange and greater involvement in informal reciprocal communication relationships [32, 34]. Two studies reported potential approaches to strengthen interdisciplinary collaboration [34, 36]. One qualitative study showed that GPs’ participation in tumor boards may improve collaboration with urologists and oncologists. The authors discussed whether participation in tumor boards could benefit GPs by improving their integration into cancer care and facilitating collaboration [34]. Another qualitative study evaluated a web-based personal electronic health record prototype (PEPA) designed to facilitate information exchange across healthcare institutions [36]. During a three-month trial, GPs who used PEPA reported improved cross-sectoral collaboration, particularly through more rapid access to and sharing of information. GPs also mentioned that PEPA could be useful for obtaining second opinions. However, the need to switch between PEPA and their own practice management system was perceived as a significant barrier [36].

#### Information needs and training requirements

Five studies addressed GPs’ sources of information and training requirements [15, 18, 25, 37, 38]. One study specified on the information needs of healthcare professionals caring for patients with cancer [18]. Another study explicitly focused on the training and information needs related to CAM [25].

According to one study, GPs feel uncertain during patient consultations due to perceived gaps in their knowledge and a lack of information [18]. Compared with oncologists and professionals working in certified cancer centers, GPs reported lower confidence in seeking information [18]. Another study reported that GPs have particular difficulty finding information on nutrition and physical activity for patients with cancer [38]. Further information needs were identified in relation to tumor therapy, CAM, and supportive therapy [18]. One qualitative study reported that GPs also perceived a need for additional training in palliative care [15].

The format and accessibility of information were also important considerations. One study found that participating GPs preferred concise, targeted information [18]. Unlike most oncologists, who prefer original research articles, GPs preferred abstracts with expert commentary, as well as German translations and abridged versions [18]. Practical experience and peer exchange were likewise considered important sources of knowledge [18]. Current medical guidelines were identified as information sources for GPs; however, one study found that German oncology guidelines were often developed without GP involvement [37]. Only a minority of guidelines explicitly identify GPs as a target audience, and many recommended diagnostic and therapeutic interventions are not applicable in primary care settings [37].

#### Need for structured programs and external support

Information on structured programs for cancer care by GPs is not available in the existing German oncological guidelines [37].

The establishment of disease management programs (DMP) for cancer care was discussed in one survey study [38]. Among participating GPs, opinions were divided: 48.3% supported the introduction of cancer-specific DMPs, whereas 51.7% opposed such programs. Moreover, 72.1% of GPs were concerned about the administrative burden associated with establishing and maintaining additional DMPs for cancer care [38].

In one survey study, participating GPs reported that bureaucratic tasks consumed substantial time [39]. They also indicated a need for more consultation time to address supportive therapies for patients with cancer, such as nutrition and physical activities [38]. Particularly in palliative care, one qualitative study highlighted the need for adequate staffing and structural support to ensure delivery of home-based care [15].

## Discussion

This scoping review provides a comprehensive overview of the role of GPs in Germany in cancer care. The included studies have shown that GPs are involved in all phases of cancer care – from diagnosis to palliative care. In addition to managing symptoms and monitoring specific therapies, GPs are considered “persons of trust”, who provide patient-centered cancer care and emotional support. They guide patients with cancer through the healthcare system and facilitate understanding of cancer diagnoses and treatment options. In contrast, organizational, functional, and system‑level integration of GPs appear comparatively weak, reflected in limited reciprocal communication, unclear responsibilities, and fragmented structures. Overall, these findings consistently suggest that GPs already make substantial contributions to cancer care through continuity of care, psychosocial support, care coordination, and the management of multimorbidity. The main challenge is therefore not the absence of a GP role, but rather the insufficient support of this role through formal organizational structures, clearly defined responsibilities, and effective communication pathways.

The findings suggest that care for patients with cancer can be improved by enabling GPs to provide more comprehensive support throughout the cancer care continuum. Continuity of care through a GP is particularly important for patients with cancer, as the focus on cancer treatment may divert attention from other chronic conditions [39, 40]. Notably, one study included in this review emphasized that healthcare utilization among cancer survivors was more strongly associated with comorbidities than with age or tumor stage [30]. However, the management of comorbidities in patients with cancer by GPs in Germany was not explicitly addressed by any of the included studies. This represents a significant gap in the current evidence base.

In line with this, none of the included studies explicitly examined the role of GPs in the care of the growing population of cancer survivors in Germany. The importance of GP involvement in survivorship care is underscored by the wide range of long-term and late effects experienced by cancer survivors, including persistent physical symptoms, psychological distress, endocrine dysfunction, and treatment-related complications [3, 41, 42]. In addition, evidence indicates that cancer survivors face an increased risk of cardiovascular morbidity and mortality compared with the general population [43, 44], highlighting the relevance of chronic disease management and preventive care within survivorship pathways. Beyond physical health consequences, survivors frequently experience fear of cancer recurrence, anxiety, depression, cognitive impairment, and substantial social and occupational challenges, with many unmet needs persisting for years after completion of treatment [45]. International survivorship frameworks increasingly advocate for shared-care models that integrate primary and specialist care [46] The findings of this review suggest that comparable structures remain insufficiently developed within the German healthcare system.

To support patients in managing their daily lives and maintaining quality of life, GPs collaborate with other healthcare providers. Effective cancer care depends on timely, concise, and reliable information exchange between professionals. The studies included in this review indicate that GPs require access to relevant clinical information and express a strong desire for greater involvement in reciprocal, bidirectional communication with specialists and other healthcare providers. However, many GPs reported dissatisfaction with existing collaboration structures. The fragmentation of medical services in Germany, combined with the strict separation between inpatient and outpatient care, is likely a major contributing factor [47]. Additionally, organ-specific specialists may lack an understanding of GP care and of the information that GPs require to provide comprehensive care for patients with cancer. As a result, important clinical information may be lost during transitions between care settings and providers. Therefore, poor collaboration poses a potential threat to patients’ health [48].

Compared to other countries such as Great Britain, GPs in Germany do not act as traditional gatekeepers. Patients in Germany have the option to contact specialists directly, without referral from a GP [49]. Consequently, patients with cancer may receive their initial diagnosis and treatment from specialist providers without any direct involvement of their GP. In some cases, GPs may not even be informed of the cancer diagnosis [50]. However, effective communication and interdisciplinary collaboration are essential for delivering holistic care to patients with cancer throughout the course of their disease. It is therefore unsurprising that patients with cancer express a preference for their GPs to be actively involved in their care [7]. Despite this, Germany currently lacks adequate structures to ensure systematic integration of GPs into the care of the growing population of patients with cancer [51]. This review showed that deficiencies in information transfer at the care interfaces frequently leave GPs without the information required to provide optimal care for their patients with cancer.

Several studies have explored strategies to improve interdisciplinary collaboration between GPs and other healthcare professionals. For example, Trabjerg et al. investigated cross-sector video consultations between patients, GPs, and oncologists in Denmark [52]. Similarly, the LoTeCaS-GP project addressed the information needs of GPs caring for long-term cancer survivors [53, 54]. Shared-care models represent a promising approach to addressing these challenges by reducing fragmentation of care and improving the coordination and quality. Such models integrate cancer-specific specialist care with generalist primary care [55]. A shared follow-up care model for patients with early-stage breast cancer has been successfully implemented in Australia, where follow-up appointments during the first five years after completion of active treatment are shared between specialists and GPs [56]. These models are usually based on structured care protocols that include follow-up schedules with clearly defined responsibilities for each provider [57]. A major advantage of shared-care models is that they incorporate the main providers of long-term follow-up care without requiring substantial additional resources. Previous research showed that shared follow-up care for patients with early-stage breast cancer was found to be more cost-effective and to reduce the workload of specialist services [56]. Implementing shared-care models could help to ensure that patients with cancer maintain regular contact with their GP while receiving coordinated specialist care. In Germany, selective contracting frameworks (e.g., § 140a, Social Code Book V) provide a suitable opportunity for testing innovative models of integrated care outside the standard system of collective contracting [58]. Thus, shared-care models could be tested as a new concept to integrating GPs into cancer care through selective contracts and, if shown to be effective, subsequently be incorporated into routine cancer care.

Overall, further research is essential to develop and implement regional and national structures that facilitate the integration of GPs into cancer care. Such efforts should be aligned with the core principles of general practice while taking into account the daily challenges of outpatient care. By 2035, Germany is projected to face a shortfall of approximately 11,000 GPs, with nearly 40% of rural regions either undersupplied or at risk of becoming undersupplied [59]. At the same time, demand for primary care services is expected to increase, further intensifying the workload of the remaining GPs and exacerbating the pressure on healthcare provision [60].

To ensure the care of patients with cancer in the coming years, efficient structures are needed for information exchange and knowledge transfer between GPs and other healthcare providers. Currently, German oncology guidelines focus predominantly on specific cancer entities and specialist-led care, with limited consideration of the role of GPs [37]. Future guideline development should therefore include explicit recommendations for the integration of GPs into cancer care pathways.

## Strengths and limitations

To our knowledge, this is the first review to compile and structure evidence on the role of GPs in cancer care in Germany. The included studies employed a wide range of methodological approaches and data sources, encompassing routine healthcare data, nationwide datasets, and the perspectives of GPs, oncologists, and other healthcare professionals. The review provides a comprehensive overview of current research in this field.

A major strength of this review is the inclusion of a broad and heterogeneous body of evidence, encompassing different study designs, data sources, and perspectives. This allowed for a comprehensive exploration of the multifaceted role of GPs in cancer care. However, this heterogeneity also constitutes an important limitation and restricted direct comparisons across studies and precluded firm conclusions. The synthesis therefore should be interpreted as providing an overview of the available evidence rather than definitive conclusions. Furthermore, no formal quality or risk-of-bias assessment was conducted, in accordance with scoping review methodology. Consequently, the findings should be interpreted as an overview of the available evidence rather than as a measure of its strength.

Our study has several additional limitations: (I) Excluding studies that focus exclusively on patient perspectives may limit insight into patient-reported experiences of GP involvement. This decision was made to maintain conceptual coherence, with patient‑focused evidence planned for a separate synthesis. (II) The search terms used were nonspecific. The terms “tumor,” “cancer,” and “oncology” are broad entry terms rather than specific MeSH terms. As the MeSH term “malignant neoplasms” is rarely used in general practice research, these broader terms were considered more appropriate for capturing relevant studies. (III) Due to access limitations, additional databases such as Embase, CINAHL, and Scopus were not searched, and relevant studies indexed exclusively in these databases may have been missed. (IV) The review focused on cancer care in general and did not specifically search for specific cancer entities. (V) We focused on studies published in indexed journals and excluded grey literature. Therefore, it is possible that the review may have overlooked recent developments on this topic. (VI) This review aimed to provide an overview of GP involvement in cancer care in Germany. As healthcare systems vary considerably between countries, the transferability of these findings to other settings is limited. In particular, the role of GPs in cancer care might vary across countries due to differences in their integration into healthcare systems, patterns of interdisciplinary collaboration, professional competencies, and care pathways. (VII) Much of the available evidence was qualitative or descriptive in nature. As a result, evidence on the effectiveness of the findings of GP involvement aimed at improving care remains limited. (VIII) The evidence base was unevenly distributed across the cancer continuum. In particular, relatively little evidence was available on survivorship, follow-up care, and the management of comorbidities.

## Implications

The findings of this review highlight the central role of GPs in the care of patients with cancer and suggest several implications for clinical practice, health policy, and future research.

For clinical practice, strengthening communication and collaboration between GPs and specialist providers should be a priority. Timely exchange of relevant clinical information, clear delineation of responsibilities, and greater involvement of GPs in cancer care planning may improve continuity of care and support more coordinated, patient-centered services. Given their longstanding relationships with patients, GPs are well positioned to address psychosocial needs, manage comorbidities, and support patients throughout the cancer trajectory.

At the policy level, the findings point to a need for structures that facilitate the systematic integration of GPs into cancer care pathways. This may include improved digital communication systems, mechanisms to support cross-sectoral collaboration, and the incorporation of primary care perspectives into oncology guideline development. Innovative models of care, such as shared-care approaches between primary and specialist care, should be explored and evaluated within the German healthcare system.

For research, several important evidence gaps were identified. Future studies should investigate the role of GPs in survivorship care, the management of comorbidities in patients with cancer, and the impact of primary care involvement on patient outcomes. In addition, research is needed to develop and evaluate interventions that improve interdisciplinary collaboration and facilitate the integration of GPs into multidisciplinary cancer care.

## Conclusion

This scoping review provides the first comprehensive overview of the role of GPs in cancer care in Germany. The available evidence demonstrates that GPs are involved throughout the entire cancer trajectory, from diagnosis and active treatment to follow-up and palliative care. Beyond symptom management and the treatment of selected cancer-related conditions, GPs provide person-centered care, psychosocial support, counselling, and care coordination, thereby serving as a central point of contact for patients with cancer. Despite their important role, GPs remain insufficiently integrated into formal cancer care structures. The literature consistently highlights challenges related to fragmented communication, limited information exchange, and unclear responsibilities at the interfaces between primary and specialist care. These barriers may hinder continuity of care. The findings also highlight important evidence gaps. In particular, little is known about the management of comorbidities in patients with cancer, the role of GPs in survivorship care, and effective models for integrating primary care into multidisciplinary cancer services. Future research should focus on developing structured models of collaboration, improving information transfer between healthcare sectors, and establishing evidence-based approaches that support the involvement of GPs throughout the cancer continuum.

## Supplementary Information


Supplementary Material 1.



Supplementary Material 2.



Supplementary Material 3.



Supplementary Material 4.


## Data Availability

All data analyzed during this study are included in this published article (and its supplementary information files).
