# Supplementary material for: The role of general practitioners in German cancer care: a scoping review
Source: BMC Prim Care. 2026 Sep 2;27:326. doi: 10.1186/s12875-026-03541-w (PMC13536645; doi:10.1186/s12875-026-03541-w)
Supplement: Supplementary file 1 [file 12875_2026_3541_MOESM1_ESM.docx]

**Supplement 1: Search Strings**

| Cochrane Library | |
| --- | --- |
| Title, Abstract, Keywords | ("general practitioner*" OR (family NEXT/1 physician*) OR "family doctor*" OR "primary care" OR "family medicine") AND (cancer OR oncolog* OR tumor) AND (german*) |
| Web of Science | |
| Topic | ("general practitioner*" OR "family physician*" OR "family doctor*" OR "primary care" OR "family medicine") AND (cancer OR oncolog* OR tumor) AND (german*) |
| Pub Med | |
| Title, Abstract | ("general practitioner*" OR "family physician*" OR "family doctor*" OR "primary care" OR "family medicine") AND (cancer OR oncolog* OR tumor) AND (german*) |
