# Supplementary material for: The role of general practitioners in German cancer care: a scoping review
Source: BMC Prim Care. 2026 Sep 2;27:326. doi: 10.1186/s12875-026-03541-w (PMC13536645; doi:10.1186/s12875-026-03541-w)
Supplement: Supplementary file 2 [file 12875_2026_3541_MOESM2_ESM.docx]

**Supplement 2**

**Guiding questions to reformatted data**

| **Patients/**  **survivor** | Are there any information about patients with specific cancer entities?  What stage of cancer are the relevant patient group in (e.g., acute treatment, remission, long-term survivorship, palliative care)?  Did these patients have comorbidities (yes/no). |
| --- | --- |
| **Physicians** | Are there any characteristics of the physicians reported, e.g., professional qualifications, junior doctors? |
| **GP roles and responsibilities** | Are GPs involved in the coordination of care for patients with cancer? Are GPs involved in the assessment of therapeutic interventions for patients with cancer during active treatment? Are GPs involved in the diagnosis of cancer?  Are GPs involved in the treatment of physical symptoms in patients with cancer? Are the GPs involved in the management of treatment-related side effects and late effects (e.g., fatigue)?  Are GPs involved in managing late effects of treatment in GP practice (e.g., polyneuropathy)?  Are GPs involved in the care of patients with cancer regarding psychological aspects (e.g., fear of treatment or death, sleeplessness, sadness, restlessness, depression)? Are GPs involved in the diagnosis and treatment of mental health conditions caused by cancer or its treatment (e.g., depression, insomnia, fear)? Do GPs play a role in maintaining the quality of life of cancer patients?  Are GPs involved in palliative care for their patients with cancer? Are GPs involved in organizing specialized palliative care in the home care setting?  Are GPs involved in communicating cancer diagnoses? Are there any difficulties in GP-patient communication with cancer patients reported? Are there any communication strategies used by GPs when communicating with cancer patients reported?  Do GPs provide advice on tertiary prevention? Do GPs provide medical social services?  Are GPs involved in the care of the relatives of cancer patients (e.g., psychological help, managing interdisciplinary teams to help relatives provide care for patients at home)? |
| **Interdisciplinary collaboration** | Are there any reported information on interdisciplinary collaboration/involvement or the provision of services to oncologists/clinicians? Do GPs have to do “legwork” for practice-based oncologists or clinicians (e.g., performing blood tests for special parameters in home care settings; organizing transportation to specialized examinations (e.g., radiological examinations))? |
| **Information sources** | Which information sources do GPs use to get information on cancer, specific therapies, or cancer-related patient issues? |
| **Barriers, facilitators, and unmet needs for GP involvement in cancer care** | What are the GPs’ needs to improve their treatment strategies (e.g., better knowledge, stronger interdisciplinary exchange/collaboration, special training)? How can GPs provide adequate care for patients? What are the needs of patients and long-term cancer survivors regarding their care provided by GPs? |
