# Supplementary material for: The role of general practitioners in German cancer care: a scoping review
Source: BMC Prim Care. 2026 Sep 2;27:326. doi: 10.1186/s12875-026-03541-w (PMC13536645; doi:10.1186/s12875-026-03541-w)
Supplement: Supplementary file 3 [file 12875_2026_3541_MOESM3_ESM.docx]

**Supplement 3**

n.i. = no information

CAM = Complementary and alternative medicine

**Outcomes**

|  | **GP roles and responsibilities** | |
| --- | --- | --- |
|  | tasks | competences |
| Boesenecker_2022 | GPs rated nutrition (N) and physical activity (PA) as (very) important both during and after cancer therapy  Two thirds of GPs viewed themselves as primary reference persons to address N and PA  GPs reported to see cancer patients on a regular basis, but hardly had access to the patient during active treatment | n.i. |
| Dahlhaus_2015 | GPs mainly respond to patients' requests for alternative treatments but do not proactively advise/ask about the use of alternative treatments.  Some refer patients to experienced CAM providers, while others only recommend what they know (e.g., vitamins).  GPsrely on personal experience in daily practice, as there is no comprehensive knowledge of CAM | GPs felt giving patients hope, supporting them (by means of affirming CAM use), and maintain trust in relationship’  GPs described own role as providing ‘spoken medicine’ in contrast to specialist care |
| Engler_2017(1) | GPs are rarely integrated into treatment decisions; GPs are not involved in discussions about which physician shall perform which therapeutic step for which reason.  other physicians informed GPs about duties they wanted GPs to perform  duties mostly included performing concomitant tests during and after active treatment, writing prescriptions and transmitting diagnoses | n.i. |
| Engler_2017(3) | n.i. | Oncologists view: GP in amicable sphere of caring & life coaching; in contrast to oncologists’ sphere of evidence-based treatment decisions; GP acceptable co-counsellor in decision-making  contrast in doing and talking  concept GP person of trust: complement to oncologists’ role as specialists, based upon their knowledge of the medical history and family situation of their patients, as well as the duration of their relationships with patients  GP persons of trust: deal with anxious patients, psychosocial problems, explain to patients what was said during oncologists’ consultations |
| Poss-Doering_2018 | n.i. | n.i. |
| Siebenhofer_2014 | n.i. | n.i. |
| Dahlhaus_2013 | GPs face numerous complex challenges and limitations when caring for palliative patients  GPs proactively coordinate care for their palliative cancer patients (use of trusted home-based care providers, emergency services, SPCO teams)  GPs reaching their limits when providing care for palliative patients (e.g., when symptoms require invasive procedures for adequate symptom control)  GPs offer home-based care; GPs commit to providing care around-the-clock; risk being overburdened if they have to care for several of these patients simultaneously  GPs involvement in palliative care increased towards end-of-life | GPs feel an attachment to their patients & want to remain involved during the palliative care phase  GPs must assess the ability of family members to serve as caregivers: Family caregivers' emotional involvement (e.g., fear, sadness, own medical disabilities) can make providing care a demanding task.  GPs feel they provide emotional support to palliative cancer patients and their families (talk about death and fear) |
| Dahlhaus_2014 | frustration about lack of care coordination between GPs and clinicians; resulting inconsistencies in care and additional time needed to explain setbacks to her patients  GP involvement changes over the course of the disease: GPs not particularly involved in curative & palliative therapy but in palliative home-based care. | GPs demarcated own role in comparison specialists’ role’:  focus more quality-of-life, when deciding on further treatment for cancer & co-morbidities, bear prognosis, burden and side effects of the treatment  fundamental role in accompanying patient throughout the whole course of cancer care,  GPs know most of cancer patients for many years: personal relationships with patients & familiarity with social & familial environments.  In addition: bio-medical knowledge of patients’ co-morbidities, health history, understanding relevance of these factors to the provision of patient-centered care  GPs help patient to understand his diagnosis and its consequences for care.  GPs want to be a resource for patients, they can ask questions and receive clarification  GPs provide form of cancer care that is less disease- /treatment-focused & more concerned with symptoms and psychosocial dimensions |
| Blaschke_2019 | SOPC is prescribed by GPs in over 90% of cases | n.i. |
| Bossert_2021 | Initial diagnosis in the outpatient sector most often by GPs | n.i. |
| Ciarlo_2016 | GPs significantly more often consulted on issues such as mental problems, complementary medicine, survivorship, palliative medicine, physical activity, physiotherapy | n.i. |
| Engeser_2016 | GPs use entire range of analgesics, co-analgesics, and adjuvants for pain control | n.i. |
| Engeser_2021 | Over 80% of palliative cancer patients treated by their GP, about 18% additionally referred to SOPC team | n.i. |
| Gremke_2023(1) | Early-onset breast cancer decreased in general practices between 2010 and 2022 | n.i. |
| Gremke_2023(2) | Compared with gynecologists, the initiation of tamoxifen therapy by GPs and oncologists leads to an increased risk for therapy discontinuation | n.i. |
| Kaiser_2020 | majority of GPs considered co-care of tyrosine kinase inhibitors (TKI) patients to be possible  GPs feel unsure/not very confident in dealing with and monitoring the progress of TKI therapies | n.i. |
| Kaiser_2022 | Hospice residents most often treated by GPs and/or palliative medicine physicians | n.i. |
| Klein_2018 | Using Complementary and alternative medicine (CAM): relaxation techniques/meditation, herbal drugs, nutritional supplements, vitamins and trace elements, homoeopathy, mistletoe therapy  To treat: fatigue, tumor-related pain, psychological (e.g., anxiety, depression), nausea and vomiting, lack of appetite and changes in sense of taste, mucosal inflammations | n.i. |
| Kone_2018 | n.i. | n.i. |
| Kostev_2017 | In terms of diagnosis, patients aged between 41 and 70 years, are particularly likely to be diagnosed by GPs  most common cancers diagnosed by GPs are cancers of the digestive organs, breast cancer, and skin cancer | n.i. |
| Kowalski_2017 | n.i. | n.i. |
| Riess_2022 | GPs are main providers of care for outpatients with cancer-associated thrombosis & issue most prescriptions for anticoagulants  selection of anticoagulants is not evidence-based and does not correspond to the approval status of the investigation period |  |
| Thong_2021 | colorectal cancer survivor: comorbidities rather than older age were associated with higher numbers of non-cancer-related GP visits | n.i. |
| Walter_2018 | lung cancer patients receive same kind of supportive care at end-of-life regardless whether they live in a rural area or not  factors for high/low utilization of healthcare: mostly age, comorbidities, and prior anticancer treatment  receiving supportive care mostly predicted by male gender & disease stage at diagnosis. | n.i. |

|  | **Interdisciplinary collaboration** |
| --- | --- |
| Boesenecker_2022 | GP highlighted the responsibilities for following participants in cancer care: (a) oncologists, (b) rehabilitation institutions, (c) GP, (d) relatives, and (e) politics  timely communication between health care providers has been criticized  GP manly satisfied with discharge letters and phone calls (from clinicians), but up to a quarter did not receive letters or phone calls in a timely manner |
| Dahlhaus_2015 | n.i. |
| Engler_2017(1) | rare cases GPs involved in formal structures (e.g., multidisciplinary quality circles on palliative care) - reported positively on these instances of shared patient care  all physicians regarded cooperation as integral part of good cancer care; specialists’ and GPs’ ideas about tasks that constitute care differed  Oncologists & urologists’ cooperation: joint treatment decision-making, focuses on tumor treatment, shared patient care, reciprocal communication in formal and informal pathways, communication with other specialists on treatment-specific topics.  GPs, discussed cooperation: supporting their patients’ day to day lives (incl quality of life and patients’ pathways through health care system), rarely integrated into treatment decisions, formal cooperation structures rarely discussed, unidirectional communication between themselves and specialists,  GPs not part of informal reciprocal communication relationships in the way they would like and find important for their patient care; to be involved they had to be proactive and make the phone calls  GPs: physicians in medical practices in outpatient settings/clinics informed GPs about duties they wanted GPs to perform (performing concomitant tests during and after active treatment, writing prescriptions and transmitting diagnoses)  GPs cooperate for the purpose of clarifying symptoms, making appointments for patient check-ups, transmission of diagnoses, treatment of co-morbidities, palliative (home) care  With assumption of control examinations (e.g., blood tests), GPs hoped to support patients in handling circumstances by providing cancer care in the community setting and diminishing the need to travel to a specialist |
| Engler_2017(3) | GPs as persons of trust support and mediate oncologists’ treatment decisions;  GPs knowledge of patients’ overall constitution & family situation can help oncologists in reaching treatment decisions, especially on termination of treatment  Oncologists: major risk (not often) GPs’ provision of out-of-date information & in challenging oncologist-patient relationships by contesting oncologists’ treatment decisions |
| Poss-Doering_2018 | PEPA potential simplifies data sharing across care settings, fast access to structured data, optimization of care processes (e.g., share lab results, look for new reports  interpreted PEPA as a structured well-functioning documentation medium  Physicians addressed the importance of provider-patient dialog |
| Siebenhofer_2014 | n.i. |
| Dahlhaus_2013 | GPs consult specialists when lacking knowledge of most effective pain killer in a given situation, or procedures for delivering medications  GPs consult specialists help support the family of the patient (need specialist support for home-based care)  in anticipation of their absence, GPs proactively coordinate care for their palliative cancer patients (use trusted providers: home-based care, emergency services, and SPCO teams)  GPs praised SPCO teams, especially when they reached their individual limits  limited capacity of SPCO teams, sometimes being nonexistent because of bureaucratic issues within a region's health system |
| Dahlhaus_2014 | GPs want better information sharing and cooperation with other providers  frustration about the lack of care coordination between herself and clinicians, and the resulting inconsistencies in care  lack of information on their patients makes it difficult to provide patient-centered care: GPs requires calling the patient themselves or demanding information from specialists and clinics |
| Blaschke_2019 | SAPV was prescribed by GPs for over 90% of the 3 cancer diagnoses (Mamma-Ca, Prostate-Ca, Colon-Ca) |
| Bossert_2021 | n.i. |
| Ciarlo_2016 | n.i. |
| Engeser_2016 | n.i. |
| Engeser_2021 | Over 80% of palliative cancer patients treated by their GP, about 18% additionally referred to SOPC team |
| Gremke_2023(1) | n.i. |
| Gremke_2023(2) | n.i. |
| Kaiser_2020 | GPs are generally motivated to provide TKI therapy for cancer patients.  GPs feel not very confident in dealing with & monitoring the progress of TKI therapies  GPs in urban areas referred significantly more patients to organ-specific specialists for TKI therapy-monitoring than GPs in rural areas  Routine treatment monitoring of TKI patients by GPs is currently hardly feasible |
| Kaiser_2022 | n.i. |
| Klein_2018 | n.i. |
| Kone_2018 | GPs’ opinions on cooperation with outpatient oncology specialists, physicians in hospitals, home care services, outpatient psycho(onco)logists/psychotherapists, hospice/palliative care units and specialized palliative home care.  overall GPs were rather or completely satisfied, except for two groups: physicians at relatively large hospitals, outpatient psycho(onco)logists/psychotherapists  home care services are rated most highly  communication and the transfer of information were rated worse, the bigger the health care institution  GPs rated other health care providers as generally competent  GPs satisfaction with cooperation: communication of sufficient information and in good time contributes strongly towards, quickly organize an appointment, health care provider is competence, received advice upon request, emotionally unburdened;  GP practice characteristics related to satisfaction with cooperation: additional qualification in palliative care was negatively associated with GPs’ satisfaction, working in a group practice was a positive predictor |
| Kostev_2017 | n.i. |
| Kowalski_2017 | From the perspective of GPs, there is no difference in communication between certified centers (breast cancer centers and colon cancer centers) and standard clinics |
| Riess_2022 | n.i. |
| Thong_2021 | n.i. |
| Walter_2018 | n.i. |

|  | **Information sources** | **Barriers, facilitators, and unmet needs for GP involvement in cancer care** |
| --- | --- | --- |
| Boesenecker_2022 | internet as information source, but differing opinions on its reliability  other sources on nutrition/PA information: medical journals, guidelines | more time during cancer patient consultation addressing nutrition and physical activities (PA)  GPs spend too many resources [e.g., time, personnel,  material] on bureaucratic efforts  GP unfamiliar with specific information on nutrition & PA in cancer patients, or where to find such information.  differing opinions on implementing structured treatment programs such as Disease Management Programs (DMP) (either half advocating or rejecting proposal of establishing DMP for cancer entities  need of DMP for follow up of cancer patients |
| Dahlhaus_2015 | n.i. | Need for More Education and Information on CAM |
| Engler_2017(1) | n.i. | Stronger involvement of GPs in cancer care  Need: sufficient information  need to establish effective ways of co-working: formal spaces of cooperation e.g., tumor boards and following evolved relationship fostered informal communication pathways like telephone calls |
| Engler_2017(3) | n.i. | n.i. |
| Poss-Doering_2018 | n.i. | n.i. |
| Siebenhofer_2014 | None of the included guidelines contained information/recommendations on structured programs for the primary care of oncology patients  8 of 21 guidelines: GPs involved in the development  4 of the 21 guidelines: GPs explicitly addressed  there are no GP care programs in oncology guidelines for Germany  most recommendations from guidelines on therapeutic or diagnostic measures cannot be implemented directly by the GP | n.i. |
| Dahlhaus_2013 | n.i. | need for special skills in end-of-life care  structural and personal support GPs need to provide home-based palliative care |
| Dahlhaus_2014 | GPs want better information sharing  GPs proactively seek information: calling the patient themselves or demanding information from specialists and clinics | n.i. |
| Blaschke_2019 | n.i. | n.i. |
| Bossert_2021 | n.i. | n.i. |
| Ciarlo_2016 | frequently used congresses and symposia  GPs were less interested in journals, but more often in textbooks  colleagues as a reliable source of information  male physicians and younger professionals search more often online than female physicians  Elderly GPs preferred to gather information from scientific journals  Time spent on information seeking: oncologists and GPs invested 0–30 min per week  Preferred source and types of information:  GPs accepted “mobile devices” as well as the “Internet”, currently used sources “experience and knowledge of (their) colleagues.”, GPs preferred German translations with abridged versions | GPs need for information on aftercare and palliative care  Feelings of uncertainty during consultation (female physicians, women felt significantly more insecure)  Reasons for uncertainty: GPs lack of knowledge, Lack of data  PGs evaluated the available information: significantly more often than oncologists chose “satisfactory” and “sufficient”  Familiarity with the task of information seeking: 42.9% of GPs chose “satisfactory.”, Oncologists and “others” working in a certified hospital significantly more often chose “very good” and “good” than GPs  Additional information needs for specific topics: Most important tumour therapy, CAM, supportive therapy  additional information on drugs (oncologists 18.4%, GPs 26.5%), supportive therapy (oncologists 16.3%, GPs 32.7%)  GPs significantly more often in need of information than oncologists (e.g., on aftercare and palliative care) |
| Engeser_2016 | n.i. | n.i. |
| Engeser_2021 | n.i. | n.i. |
| Gremke_2023(1) | n.i. | n.i. |
| Gremke_2023(2) | n.i. | n.i. |
| Kaiser_2020 | n.i. | majority of participants had insufficient knowledge of TKI side effects & potential interactions of TKIs with other drugs, alternative preparations & nutritional products |
| Kaiser_2022 | n.i. | n.i. |
| Klein_2018 |  | GPs prefer information on CAM provided on “information platforms on the internet”, “lectures on specific CAM-related topics”, “regular e-mail newsletters”  GPs favor the opportunity to contact experts  List of preferred training programs |
| Kone_2018 |  | GPs general satisfaction with collaboration is strongly linked to sufficient information and timely communication |
| Kostev_2017 | n.i. | n.i. |
| Kowalski_2017 | n.i. | n.i. |
| Riess_2022 | n.i. | n.i. |
| Thong_2021 | n.i. | n.i. |
| Walter_2018 | n.i. | n.i. |
