# Supplementary material for: The role of general practitioners in German cancer care: a scoping review
Source: BMC Prim Care. 2026 Sep 2;27:326. doi: 10.1186/s12875-026-03541-w (PMC13536645; doi:10.1186/s12875-026-03541-w)
Supplement: Supplementary file 4 [file 12875_2026_3541_MOESM4_ESM.docx]

**Supplement 4**

**Characteristics**

| Author_year | Sample (n) | Gender (professionals) | Age | Experience | Town | Education/ Qualification | Practice type |
| --- | --- | --- | --- | --- | --- | --- | --- |
| Blaschke_2019 | performance datapatients n = 9,273 |  |  |  |  |  |  |
| Boesenecker_2022 | GPs n = 61 | male n = 34, 56%  female n = 27, 44% | ranging from 33 to 74 years (mean age 53.71±9.26 years) | more than 20 years (46.0%), 31.1% between 10 and 19 years, and 23.0% for less than 10 years |  |  |  |
| Bossert_2021 | performance data  patients n = 13,111 |  |  |  |  |  |  |
| Ciarlo_2016 | oncologists n = 147 GPs n = 147 others n = 201 | Oncologists: Male n = 112, 76% Female n = 35, 24%  GPs:  Male n = 89, 61%  Female n = 54, 37%  Other  Male n = 110, 55%  Female n = 89, 45% | GPs: mean age 44.3 years, Age (%):  31–40: n = 11 (7.48),  41–50: n = 43 (29.25),  51–60: n = 44 (29.93),  61–70: n = 23 (15.65), >71: n = 3 (2.04),  No indication: n = 23 (15.65) |  |  |  |  |
| Dahlhaus_2013 | GPs n = 13 | female n = 6, 46%) male n = 7, 54%) |  | mean GP working experience of 16.6±8.8 years (n = 12, one GP made no specification) | rural town (less than 5,000 inhabitants)  n = 2, provincial town (5,000 to 20,000 inhabitants) n = 3, medium-sized town (20,000 to 100,000 inhabitants) n = 6, large town (above 100,000 inhabitants) | general medicine n = 11, internal medicine n = 2. additional qualification: palliative medicine n = 7, psychotherapy n = 1 | joint practice n = 6, single-handed practice n = 7 |
| Dahlhaus_2014 | GPs n = 30 |  |  | work experience: from 3 to 41 years, median 13.5 years  (n = 30) | rural town (less than 5000 inhabitants) n = 7, provincial town (5000–20000 inhabitants) n = 2, medium sized town (20000–100000 inhabitants) n = 12, large town n = 9 |  | joint practice n = 13, single-handed practice n = 17 |
| Dahlhaus_2015 | GPs n = 10 | male n = 4, 40% female n = 6, 60% |  |  | rural n = 3, large town n = 3, medium-sized town n = 4 | additional certification: physical therapy/naturopathic medicine/palliative care n = 1, nutrition  n = 3 | joint practice n = 5, single handed practice n = 5 |
| Engeser_2016 | performance data: 9752 GP prescriptions for 1362 patients |  |  |  |  |  |  |
| Engeser_2021 | performance data patients n = 3,535 |  |  |  |  |  |  |
| Engler_2017(1) | GPs n = 42  urologists n = 21  oncologists n = 21 | female GPs n = 19, 45%  female urologists  n = 2, 10%  none of the oncologists |  | on average, the GPs (n = 42) had been working for 15.2 ± 9.8 years in general practice, oncologists (n = 19) for 10.5 ± 6.5 years, and the urologists (n = 21) for 13.6 ± 8.7 years each in their own medical practice |  |  |  |
| Engler_2017(3) | oncologist n = 15 | female n = 3  male n = 12 | median age 58 years, ranging from 35 to 62 | median work experience was 22.5 years, ranging from 1 to 30 years |  | 8 interviewees reported having additional training in palliative care, two reported additional training in psycho-oncology or psychotherapy and two in infectiology. |  |
| Gremke_2023(1) | performance data patients n = 2,359 |  |  |  |  |  |  |
| Gremke_2023(2) | database  patients n = 284,383 |  |  |  |  |  |  |
| Kaiser_2020 | GPs n = 553 | male n = 295, 54% female n = 248, 46% | < 30 n = 38 (7%)  30-40 n = 1 (0,2%)  40-50 n = 143 (26%) 50-60 n = 203 (37%) 60-70 n = 146 (26%) >70 n = 14 (2%) | private practice n = 283 (52%)  joint practice n = 264 (48%) | rural town n = 114 (21%)  Small town n = 103 (19%)  Medium-sized town  n = 106 (19%)  Large city n = 226 (41%) | medical specialists for:  general practice  n = 436 (81%), internal medicine n = 73 (14%), practical physician (education outdated) in n = 29 (5%) |  |
| Kaiser_2022 | hospizes n = 112 |  |  |  |  |  |  |
| Klein_2018 | final sample size  n = 1,049  GPs n = 209  medical specialists  n = 437  oncology nurses and medical assistants  n = 159  psychologists and social workers engaged in cancer care n = 244 | female 55% | mean age 53±8 years | years of work experience mean 20±9 |  |  |  |
| Kone_2018 | GPs n = 1,428 | male n = 823  female n = 549 |  | work experience:  Up to 10 years n = 255 (20),  >10 up to 20 years  n = 355 (28),  >20 up to 30 years  n = 507 (39),  >30 up to 40 years  n = 151 (12),  >40 up to 50 years  n = 16 (1) | rural/provincial town n = 781, medium-size/large town n = 642 | additional qualifications: palliative care n = 227 (16%), pain management n = 76 (5%), Psychotherapy n = 267 (19%), CAM (including acupuncture, homeopathy, naturopathy) n = 520 (36%) | practice type:   single practice n = 702, 51%,  group practice  n = 677, 49% |
| Kostev_2017 | performance data patients n = 31,628 |  |  |  |  |  |  |
| Kowalski_2017 | GPs n = 200 |  |  | established since:  less than 10 years 34 (17%)  10 years or more 166 (83%) | population of location: village (≤2000) n = 22 (11%)  rural town (2001 to 5000) n = 28 (14%)  small town (5001 to 20,000) n = 51 (26%)  medium-sized town (20,001 to 100,000) n = 43 (22%)  large city (100,001 to 1 million) n = 42 (21%)  city with over a million inhabitants (≥1 million) n = 14 (7%) | medical specialists for:  GP n = 166 (83%),  practical physician (education outdated) n = 11 (6 %)  internal medicine n = 23 (12%) |  |
| Poss-Doering_2018 | gastrointestinal cancer patients n = 11  GPs n = 3 | GPs female n = 3, 100% | age range  GPs = 29 - 58 |  |  |  |  |
| Riess_2022 | performance data patients n = 7,313 |  |  |  |  |  |  |
| Siebenhofer_2014 | medical guidelines  n = 559 |  |  |  |  |  |  |
| Thong_2021 | performance data survivors n = 1,718 |  |  |  |  |  |  |
| Walter_2018 | performance data patients n = 12,929 |  |  |  |  |  |  |

GP = General Practitioner
